# Self-Reported Fall-Related Injury and Its Associated Factors among Adults with Visual Impairment Attending St. Paul's Hospital Millennium Medical College, Addis Ababa, Ethiopia

**DOI:** 10.4314/ejhs.v33i2.11

**Published:** 2023-03

**Authors:** Yingesu Lemma Mengste, Gizachew Tilahun Belete, Biruk Lelisa Eticha, Tarekegn Cheklie Zeleke

**Affiliations:** 1 St. Paul's Hospital Millennium Medical College, Department of Ophthalmology, Addis Ababa, Ethiopia; 2 University of Gondar, School of Medicine, Department of Optometry, Gondar, Ethiopia

**Keywords:** Fall, Visual impairment, Addis Ababa, SPHMMC

## Abstract

**Background:**

Falls account for vast majority of fractures and are a significant reason for trauma related hospital admissions. The main aim of this study is to determine the prevalence of self-reported fall, related injuries, and associated factors among adult patients with visual impairment.

**Methods:**

Hospital-based cross-sectional study was conducted from July to August 2021. Systematic random sampling technique was used. The data were entered into Epi-data version 3.1 and exported to SPPS version 26 for analysis. Frequency, mean, and percentage, were used to summarize the descriptive data. The association between the outcome variable and explanatory variables was assessed using binary and multivariate logistic regressions. The adjusted odds ratio was calculated, and variables with a p-value below 0.05 at the 95% confidence interval (CI) were considered statistically significant.

**Result:**

A total of 487 study participants were involved in this study with a response rate of 93.83%. The mean age of the study participants was 52 ± 16.26 years. The overall prevalence of self-reported fall was 36.1 %. Being female, being older than 64 years of age, rural residence, fear of falling, and blind stage of visual impairment were significantly associated with falling.

**Conclusion:**

The prevalence of self-reported fall was high among visually impaired individuals. Female sex, age more than 64 years, rural residence, fear of falling, and blind stage of visual impairment were significantly associated with falling. Reducing patients' chances of suffering from falling-related injuries and consequences requires raising awareness about the burden, danger, and effects of falling on persons who are visually impaired.

## Introduction

Fall is defined as “a sudden and unintentional change of posture to the ground or a lower level, onto an object, floor, pavement, ground, or any other type of surface” (1). It is a significant issue for public health and medicine. Falling significantly lowers quality of life and can cause fear of falls, decreased physical activity, performance decline, loss of daily living activities, social isolation, depression, and an elevated risk of subsequent falling (2).

Falls are one of the main sources of injury because they expose the body to excessive energy, which results in physical harm (3). Serious injuries, such as acute head trauma, significant lacerations, or fractures, occur in five to ten percent of falls (4). Falls account for about 90% of fractures and are a significant reason for trauma related hospital admissions (5). It also accounts for 40% of all injury deaths (6) likewise injury is among the top 10 causes of death and Approximately, 90% of injury-related mortality occurred in low and middle-income countries (7, 8). Falls-related injuries cost money for medical professionals, social services, patients, and their families (9). The anticipated annual medical expenditures for falling in 2015 were 50 billion US dollars (10).

People with visual impairment have an increased risk of falling (11–13). Visual impairment (VI) is a severe health issue that significantly affects a person's personal, professional, and social life (14). The World Health Organization estimates that there are over 314 million visually impaired persons worldwide, whereas the prevalence of low vision is 3.7% in Ethiopia (14, 15). Individuals with VI are about two to three times more likely to fall, experience recurrent falls, and sustain fractures than people without VI (16, 17).

Numerous research conducted worldwide shown that the percentage of visually impaired people who fall ranges from 10% to 46% (17–21). Age, gender, the degree of vision impairment, psychosocial variables, the physical surroundings, fall anxiety, depression, concurrent medical conditions, poly-medications, and pre-existing medical comorbidities are just a few of the many associated factors for falling (1, 22, 23).

In order to create preventive measures that can be incorporated into prevention programs, it may be helpful to examine modifiable associated factors like Visual impairment (12, 24).

There were few pieces of evidence about the frequency of falls among visually impaired people in Ethiopia. The prevalence of self-reported falls, related injuries, and associated factors among people with visual impairment is thus estimated in part by the findings of this study. Finally, the ultimate objective of this research is to determine the prevalence, related injury, and associated factors of self-reported fall among adult patients with visual impairment attending St. Paul's Hospital Millennium Medical College, Addis Ababa, Ethiopia, 2021.

## Methods and Materials

**Study design and setting:** The study was carried out at St. Paul's Hospital Millennium Medical College (SPHMMC) from July to August 2021 utilizing a hospital-based cross-sectional study design. The facility has sub-specialty clinics with the tools necessary to examine a patient with eye problems. For the approximately 6 million residents of Addis Ababa and the surrounding areas, it acts as the last point of referral.

In the city, there are two hospitals with tertiary eye care facilities and six hospitals with secondary eye care centers. Secondary eye care facilities refer patients with eye disorders to such tertiary facilities; SPHMMC is one of those tertiary facilities and sees roughly 4000 outpatient patients a month.

**Sample size determination**: The sample size was determined by using single population proportion formula taking the prevalence of falling was taken from a prevalence study conducted in Gondar, Ethiopia,(26) which was 26.8% (0.268); 1 − P = 0.732; d, a margin of error 4% = 0.04, and the sample size was 471. Using Epi-info software, the sample size was estimated with associated factors in mind. Taking systemic comorbidity and medication use as the two primary consistent predictors for fall, the results were 178 and 61, respectively. As a result, the sample size chosen for the prevalence was sufficiently bigger than the two, and when the 10% non-response rate was taken into account, the final sample size was 519.

**Sampling technique and procedure**: The samples were selected using systematic random sampling from all patients attending St. Paul's Hospital Millennium Medical College at the Department of Ophthalmology. According to information from triage offices, every day, 200 individuals receive ocular medical care, of which 175 are visually impaired.

As a result, there are 1400 study participants overall during the research time. The k value for systematic random sampling was 3 (1400/519). The first sample was chosen at random from a group of one to three participants, and then each subsequent sample was amused.

**Data collection tool, procedures, and Analysis**: Three trained optometrists who participated in the study used a self-administered structured questionnaire, a clinical examination, and a data extraction checklist to collect the data. Data on the socio-demography and fall experience of the study participants were gathered using the self-administered questionnaire. While the scope of the clinical examination ranges from determining the monocular distance visual acuity of study participants using a projected Snellen chart calibrated for the distance of the examination room at three meters to weight and height measurements using a digital balance and meter, respectively. The remaining data including primary diagnosis confirmed by a physician (as primary causes for visual impairment) were extracted from medical charts using a data extraction checklist. Falling was considered when a patient is coming to rest unintentionally on the ground or another lower-level one or more times during the past 12 months (27) whereas the severity of visual impairment was measured based on presenting visual acuity in the better eye (28).

After being entered into Epi-data version 3.1, the acquired data was exported to SPPS version 26, where it was then analyzed. Descriptive statistics such as frequency, mean, percentage, and standard deviation were used to summarize the descriptive part of the data. The relationship between falling and various independent factors was determined using binary logistic regression, and the model's fitness was assessed using Hosmer and Lemeshow's goodness of fit test. To find statistically significant factors, variables having a p-value of less than 0.2 at bivariable logistic regression were included to a multivariable logistic regression. With multivariable binary logistic regression, a P-value of 0.05 or less was deemed significant.

**Ethical consideration:** The University of Gondar, College of Medicine and Health Sciences ethics review committee granted ethical clearance prior to the study's execution with the reference number (684/06/21). Additionally, SPHMMC gave approval for the data gathering to continue. Each participant was given the opportunity to give their oral consent after being informed of the study's goals and their complete ability to stop or decline participation. The World Medical Association Declaration of Helsinki was followed in conducting the study. By eliminating identifiers from the data gathering process, protecting their privacy, and storing the data securely, the confidentiality of the information was carefully maintained.

## Results

**Socio-demographic characteristics of the study participants**: A total of 487 study participants were involved in the study with a response rate of 93.83%. The mean age was 52 ±16.26 years. Of all participants, 200(41.1%) were between the ages of 45 and 64 years. Among study participants, 233(47.8%) were male and about 353(72.5%) were urban residents. About 154(31.6%) study subjects had completed secondary school and among all study participants, 152(31.2%) were housewives in their occupation. One hundred sixty-two or 33.3% of participants earned between 1600 and 3000ETB per month. About 66.3% of the participant's BMI category was normal (18.5–24.9 kg/m^2^) followed by overweight (>25 kg/m^2^) (22.8%) (see Table 1).

**Table 1:** Socio-demographic characteristics of study participants at SPHMMC, Addis Ababa, Ethiopia, 2021

Variable	Frequency (%)
Age	
18–44	153 (31.4)
45–64	200 (41.1)
>64	134 (27.5)
Sex	
Male	233 (47.8)
Female BMI (kg/m^2^)	254 (52.2)
(18.5–24.9)	323 (66.3)
(<18.5)	53 (10.9)
(>25)	111 (22.8)
Educational status	
Can't read and write	63 (12.9)
Read and write	62 (12.7)
Primary school	79 (16.2)
Secondary school	154 (31.6)
College and above	129 (26.5)
Occupation	
Housewife	152 (31.2)
Merchant	64 (13.1)
Employed	135(27.7)
Unemployed	22 (4.5)
Farmer	46 (9.4)
Retired	23 (4.7)
Others	45 (9.2)
Residence	
Urban	353 (72.5)
Rural	134 (27.5)
Monthly income	
<1600	113 (23.2)
1600–3000	162 (33.3)
3001–4000	96 (19.7)
>4000	116 (23.8)

**Eye and vision-related conditions of the study participants**: Based on the better eye's presented visual acuity, the level of visual impairment was determined. Considering that 43 (8.8%) of the study's participants were blind and had vision worse than 3/60, Cataract was the primary cause of visual impairment in 142 (29%) of subjects. The majority of patients had eye treatments (see Table 2).

**Table 2:** Eye and vision-related conditions of study participants attending SPHMMC, Addis Ababa, Ethiopia, 2021

Variable	Frequency (%)
Degree of visual impairment	
Mild	161 (33.0)
Moderate	254 (52.2)
Severe	29 (6.0)
Blindness	43 (8.8)
Cause of visual impairment	
Cataract	142 (29.2)
URE	109 (22.4)
Glaucoma	84 (17.2)
AMD	15 (3.0)
Diabetic retinopathy	29 (6.0)
Others**	108 (22.2)
Use of any treatment for eye	
Yes	390 (80.1)
No	97 (19.9)
Type of treatment used	
Medication	198 (40.7)
Surgery	86 (17.7)
Laser	20 (4.1)
Spectacle	86 (17.7)

URE-Uncorrected Refractive Error, AMD-Age-Related Macular Degeneration

Others**-trachoma, keratitis, hypertensive retinopathy

**Behavioral and general health-related conditions of the study participants:** Among study participants, almost two-thirds of them, 323(66.1%) were non-alcoholic, only a few participants 44(9.0%) were smokers and 64.3% have caregivers. Almost half of study subjects 250(51.3%) in the study were not concerned about falling. While 97(19.9%) were very concerned and the remaining 140(28.7%) were somewhat concerned about falling.

Among study subjects, almost all 426(87.5%) move to a certain place by him/herself and about 404(83.3%) didn't work regular physical exercise. One hundred sixty-six (34.1%) were suffered from systemic co-morbidities, and they were all taking medication (see table 3).

**Table 3:** Fall experience of study participants attending SPHMMC, Addis Ababa, Ethiopia, 2021

Variables	Frequency (%)
Means of mobility	
Guided by another person	19 (3.9)
Walking with aid	40 (8.2)
By him/herself	426 (87.5)
Physical exercise	
Yes	83 (17.0)
No	404 (83.3)
Systemic health problem	
Yes	166 (34.1)
No	321 (65.9)
Types of systemic health problems	
Diabetic	76 (15.6)
Hypertension	74 (15.2)
Others	16 (3.3)
Systemic medication intake	
Yes	166 (34.1)
No	321 (65.9)
Kind of systemic medication	
Antihypertensive drug	74 (15.2)
Antidiabetic	76 (15.6)
Others	16 (3.3)
Falling history	
Yes	176 (36.1)
No	311 (63.9)
Frequency of falling	
One	48 (27.3)
Two	43 (24.4)
Three and above	85 (48.3)
Injury from fall	
Yes	56 (31.8)
No	120 (68.2)
Type of injuries(n=56)	
Bruising/abrasion/skin tear	15 (26.7)
Fracture/dislocation	14 (25.0)
Back pain	14 (25.0)
Bleeding	10 (17.8)
Loss of consciousness	1 (1.8)
Head injury	1 (1.8)
Others	1 (1.8)
Perceived cause for falling	
Reduced vision	103 (58.15)
Muscle weakness	21 (11.9)
Inconvenient road	25 (14.2)
Others	27 (15.3)

**Fall experience among study participants:** The prevalence of self-reported fall among visually impaired individuals was 36.1% (CI: 32.0, 40.2). Among visually impaired participants, 48(27.3%) reported one fall, 43(24.4%) reported two falls and the remaining 85(48.3) experienced three and more falls. Among visually impaired participants who reported falling, 56(31.8%) suffer some sort of injury. Self-reported falls typically resulted in bruising/abrasion/skin tears (26.7%) and bleeding (17.8%) injuries. Of those who have fallen, more than half (58.5) thought that a loss of vision was the reason they did so. Twenty one (11.9%) thought of muscle weakness they had, while 25 (14.2%) thought of an uncomfortable road, and the remaining thought of other causes for falling (Table 3).

**Factors associated with falling among visually impaired**: According to bivariable logistic regression, a number of factors, including sex, age, body mass index, education level, place of residence, monthly income, drinking behavior, fear of falling, and degree of vision impairment, were associated with self-reported falls. Sex, age, place of residence, fear of falling, and degree of visual impairment were, nonetheless, significantly associated with falling according to multivariable logistic regression analysis.

Female study subjects were 2 times (AOR=2, 95% CI: 1.19–3.38) more likely to fall in comparison to male subjects. Those older than 64 years were 3 times (AOR=3.03, 95% CI: 1.43–6.43) more likely to experience falling than those whose ages were between 18 and 44 years. The odds of fall among participants lived in rural areas were 1.69 times (AOR=1.69, 95% CI: 1.03–2.77) more than those who live in urban areas. Those participants who had a concern about falling were 5.17 times (AOR=5.17, 95% CI: 2.70–9.90) more likely to fall as compared to participants not concerned about falling. The odds of fall was 4.20 times (AOR= 4.20, 95% CI: 2.70–9.90) higher in study subjects with blind stage of visual impairment than mildly visual impaired (see Table 4).

**Table 4:** Associated factors of falling among visually impaired patients attending SPHMMC, Addis Ababa, Ethiopia, 2021

Variables	Falling		COR	AOR
	
	Yes	No		
Sex				
Male	70	163	1.00	1.00
Female	106	148	1.67(1.15,2.43)	2.00(1.19,3.38) *
Age				
18–44	36	117	1.00	1.00
45–64	71	129	1.79(1.12,2.87)	1.87(1.03,3.41)
>64	69	65	3.45(2.08,5.71)	3.03(1.43,6.43) *
BMI				
Normal	126	197	1.00	1.00
Underweight	10	43	0.36(0.18,0.75)	0.56(0.24,1.31)
Overweight	40	71	0.88(0.56,1.38)	0.66(0.38,3.33)
Educational status				
Can't read and write	41	22	7.38(3.77,14.48)	1.34(0.54,3.33)
Read and write	24	38	2.50(1.28,4.88)	0.74(0.31,1.77)
Primary school	34	45	2.99(1.61,5.56)	1.48(0.70,3.11)
Secondary school	51	103	1.96(1.14,3.39)	1.16(0.60,2.22)
College and above	26	103	1.00	1.00
Monthly income				
<1600	54	59	1.61(0.95,2.74)	0.87(0.43,1.71)
1600–3000	58	104	0.98(0.60,1.61)	0.71(0.38,1.33)
3001–4000	22	74	0.52(0.89,0.96)	0.59(0.28,1.24)
>4000	42	74	1.00	1.00
Residence				
Urban	113	240	1.00	1.00
Rural	63	71	1.89(1.26,2.83)	1.69(1.03,2.77) *
Drinking habit				
Non-alcoholic	98	225	1.00	1.00
Previously alcoholic	37	25	3.40(1.94,5.95)	1.88(0.94,3.75)
Currently alcoholic	41	61	1.54(0.97,2.45)	0.97(0.54,1.72)
Fear of fall				
Not concerned	49	201	1.00	1.00
Somewhat concerned	58	82	2.90(1.83,4.59)	1.92(1.14,3.24)
Very concerned	69	28	10.10(5.88,17.33)	5.17(2.70,9.90) **
Degree VI				
Mild	32	129	1.00	1.00
Moderate	98	156	2.53(1.60,4.02)	2.35(1.39,3.96)
Severe	17	12	5.71(2.48,13.15)	2.96(1.06,8.26)
Blindness	29	14	8.35(3.96,17.61)	4.20(1.70,10.38) *

AOR=Adjusted Odd Ratio. COR=Crude Odd Ratio

n=Sample Size. VI=visual impairment.

* P-value <0.05

** P-value<0.001

## Discussion

The current study revealed that the prevalence of self-reported fall among visually impaired adults was 36.1% (95% CI: 32–40.2). This finding was in line with the findings of studies conducted in Iran (35.7%, 33.6%) (29, 30), Thailand (37.8%) (19), Brazil (32.5%) (31), and Italy (35.9%) (32). However, the finding of this research was higher than studies from USA (27%) (4), Australia (29.8%) (33), Italy (28.6%) (34), China (10.7%) (21), Nigeria (23%) (35) and Gondar-Ethiopia (26.8%) (26). The discrepancy might be due to differences in the study population characteristics, sample size, or methodology. For instance, USA, Australia, Italy, and China are economically developed and eye care and health-related facilities are easily accessible which helps to prevent both visual impairment and falling. The Ethiopian study selected study subjects based on impairment of either eye.

On the other hand, this finding was lower than studies conducted in Australia (42.5%) (27), and USA (46.7%) (36). This difference might be due to the criteria for selecting study subjects. Their study mainly focused on older age groups, in which age is a higher risk for falling and visual impairment (37, 38). While this study includes participants whose age is 18 and above.

Regarding associated factors gender, age, residence, fear of fall, and degree of visual impairment were significantly associated with falling. Concerning gender, female study subjects were more likely to fall in comparison to male subjects. Similar results were reported from USA, Australia, Taiwan, and Nigeria (5, 20, 33, 35). It might be because of this commonality that more women than men are affected by blindness and moderate to severe vision impairment (39).

This study also discovered that participants over the age of 64 had a higher odds of fall than participants between the ages of 18 and 44. Studies undertaken in Gondar, Ethiopia, Latin America, and the USA confirm this conclusion (22, 26). This could be due to physiological changes in muscle and bone, and greater burden of visual impairment in older age (40).

Those who live in rural areas were more likely to fall as compared to those who resided in urban areas. Similarly, the study conducted in USA and Nigeria revealed that falling was higher in rural residents (35, 41). High-risk professions like farming and forestry as well as limited access to medical facilities in rural locations might be the possible reason for the result.

Fear of fall among visually impaired individuals was the other variable associated with falling in the regression model. Study subjects reported somewhat and very high concern about fall during daily living activities were more likely to fall compared to those who had not been concerned about falling. This finding was similar to studies conducted in Australia, USA, and Gondar-Ethiopia (26, 42, 43). Fear of fall affects 20–50% of old adults and may be a rational psychological response to previous falls (44). Fear of falling may have a direct effect on balance control through psychological factors such as anxiety, which leads to stiffening of joints and gait changes, which in turn, may lead to an increased risk of falling (42).

The severity of visual impairment affects an individual's ability to respond to environmental hazards. Cataract and glaucoma were the most common eye disease that causes visual impairment found in this study. Besides, blind individuals were more likely to fall than those with mild visual impairment. This finding was similar to studies conducted in USA, Australia, Taiwan, and United Kingdom (5, 20, 27, 36, 45). Contrary to this study, the degree of visual impairment was not associated with falling for studies conducted in China, Nigeria, and Gondar-Ethiopia (19, 26, 35). This might be due to methodological difference; in the Nigerian and Ethiopian studies, visual acuity was not measured objectively but rather used self-reported by participants.

The prevalence of self-reported fall was high. Factors such as age more than 64 years, female sex, rural residence, fear of falling, and blind stage of visual impairment were statistically significantly associated with falling. Reducing patients' chances of suffering from falling-related injuries and consequences requires raising awareness about the burden, danger, and effects of falling on persons who are visually impaired.

It would have been better if there was a control group to compare with because it can give better pictures of falling due to visual impairment. The questionnaire used to assess falling was based on the recalling ability of participants so it may be subjected to recall bias and also VA was taken after falling that may not reflect the actual acuity at the time of falling.

## References

[1] AL Tehewy MM, Amin GE, Nassar NW (2015). A study of rate and predictors of fall among elderly patients in a university hospital. Journal of patient safety.

[2] Taheri-Kharameh Z, Poorolajal J, Bashirian S (2019). Risk factors for falls in Iranian older adults: a case-control study. International journal of injury control and safety promotion.

[3] Fite RO, Mesele M, Wake M, Assefa M, Tilahun A (2020). Severity of injury and associated factors among injured patients who visited the emergency department at Wolaita Sodo Teaching and Referral Hospital, Ethiopia. Ethiopian journal of health sciences.

[4] Ganz DA, Bao Y, Shekelle PG, Rubenstein LZ (2007). Will my patient fall?. Jama.

[5] Patino CM, McKean-Cowdin R, Azen SP (2010). Central and peripheral visual impairment and the risk of falls and falls with injury. Ophthalmology.

[6] Rubenstein LZ (2006). Falls in older people: epidemiology, risk factors and strategies for prevention. Age and ageing.

[7] Woldemichael K, Berhanu N (2011). Magnitude and pattern of injury in Jimma University specialized hospital, South West Ethiopia. Ethiopian journal of health sciences.

[8] Abafita BJ, Abate SM, Kasim HM, Basu B (2020). Pattern and outcomes of injuries among trauma patients in Gedeo Zone, Dilla, South Ethiopia: a 5 years retrospective analysis. Ethiopian journal of health sciences.

[9] Coutinho ES, Fletcher A, Bloch KV, Rodrigues LC (2008). Risk factors for falls with severe fracture in elderly people living in a middle-income country: a case control study. BMC geriatrics.

[10] Florence CS, Bergen G, Atherly A, Burns E, Stevens J, Drake C (2018). Medical costs of fatal and nonfatal falls in older adults. Journal of the American Geriatrics Society.

[11] Dhital A, Pey T, Stanford MR (2010). Visual loss and falls: a review. Eye.

[12] de Boer MR, Pluijm SM, Lips P (2004). Different aspects of visual impairment as risk factors for falls and fractures in older men and women. Journal of bone and Mineral Research.

[13] Coleman AL, Stone K, Ewing SK (2004). Higher risk of multiple falls among elderly women who lose visual acuity. Ophthalmology.

[14] Alswailmi FK (2018). Global prevalence and causes of visual impairment with special reference to the general population of Saudi Arabia. Pakistan journal of medical sciences.

[15] Berhane Y, Worku A, Bejiga A (2007). Prevalence and causes of blindness and low vision in Ethiopia. Ethiopian Journal of Health Development.

[16] Kingston JT (2018). Visual impairment and falls: outcomes of two fall risk assessments after a Four-Week fall prevention program. Journal of Visual Impairment & Blindness.

[17] Lamoreux EL, Chong E, Wang JJ (2008). Visual impairment, causes of vision loss, and falls: the Singapore Malay Eye Study. Investigative ophthalmology & visual science.

[18] Hong T, Mitchell P, Burlutsky G, Samarawickrama C, Wang JJ (2014). Visual impairment and the incidence of falls and fractures among older people: longitudinal findings from the Blue Mountains Eye Study. Investigative ophthalmology & visual science.

[19] Pattaramongkolrit S, Sindhu S, Thosigha O, Somboontanot W (2013). Fall-related factors among older, visually-impaired Thais. Pacific Rim International Journal of Nursing Research.

[20] Kuang T-M, Tsai S-Y, Hsu W-M, Cheng CY, Liu J-H, Chou P (2008). Visual impairment and falls in the elderly: the Shihpai Eye Study. Journal of the Chinese Medical Association.

[21] Zhou H, Peng K, Tiedemann A, Peng J, Sherrington C (2019). Risk factors for falls among older community dwellers in Shenzhen, China. Injury prevention.

[22] Talbot LA, Musiol RJ, Witham EK, Metter EJ (2005). Falls in young, middle-aged and older community dwelling adults: perceived cause, environmental factors and injury. BMC public health.

[23] Verma SK, Willetts JL, Corns HL, Marucci-Wellman HR, Lombardi DA, Courtney TK (2016). Falls and fall-related injuries among community-dwelling adults in the United States. PLoS one.

[24] Omotoye OJ, Ajayi IA, Bodunde OF (2019). Factors responsible for poor visual outcome following emergency eye surgery in a tertiary eye centre. Ethiopian Journal of Health Sciences.

[25] Hodge C, Lawless M (2008). Ocular emergencies. Australian family physician.

[26] Gashaw M, Janakiraman B, Minyihun A, Jember G, Sany K (2020). Self-reported fall and associated factors among adult people with visual impairment in Gondar, Ethiopia: a cross-sectional study. BMC public health.

[27] Lamoureux E, Gadgil S, Pesudovs K (2010). The relationship between visual function, duration and main causes of vision loss and falls in older people with low vision. Graefe's archive for clinical and experimental ophthalmology.

[28] Shen H, Zhang H, Gong W (2021). Prevalence, Causes, and Factors Associated with Visual Impairment in a Chinese Elderly Population: The Rugao Longevity and Aging Study. Clin Interv Aging.

[29] Ghanbary A, Salehi Dehno N, Moslemi Haghighi F, Torabi M (2013). The prevalence and correlates of falling down in the older adults over 55 years in Shiraz. Iranian Journal of Ageing.

[30] Mortazavi H, Tabatabaeichehr M, Taherpour M, Masoumi M (2018). Relationship between home safety and prevalence of falls and fear of falling among elderly people: a cross-sectional study. Materia socio-medica.

[31] Álvares LM, Lima RdC, Silva RAd (2010). Falls by elderly people living in long-term care institutions in Pelotas, Rio Grande do Sul State, Brazil. Cadernos de saude publica.

[32] Cesari M, Landi F, Torre S, Onder G, Lattanzio F, Bernabei R (2002). Prevalence and risk factors for falls in an older community-dwelling population. The Journals of Gerontology Series A: Biological Sciences and Medical Sciences.

[33] Gill T, Taylor AW, Pengelly A (2005). A population-based survey of factors relating to the prevalence of falls in older people. Gerontology.

[34] Mancini C, Williamson D, Binkin N, Michieletto F, De Giacomi GV (2005). Epidemiology of falls among the elderly. Igiene e sanita pubblica.

[35] Bekibele C, Gureje O (2010). Fall incidence in a population of elderly persons in Nigeria. Gerontology.

[36] Crews JE, Chou C-F, Stevens JA, Saaddine JB (2016). Falls among persons aged≥ 65 years with and without severe vision impairment—United States, 2014. Morbidity and Mortality Weekly Report.

[37] Trombetti A, Reid K, Hars M (2016). Age-associated declines in muscle mass, strength, power, and physical performance: impact on fear of falling and quality of life. Osteoporosis international.

[38] Salvi S, Akhtar S, Currie Z (2006). Ageing changes in the eye. Postgraduate medical journal.

[39] Bourne RR, Flaxman SR, Braithwaite T (2017). Magnitude, temporal trends, and projections of the global prevalence of blindness and distance and near vision impairment: a systematic review and meta-analysis. The Lancet Global Health.

[40] Brundle C, Waterman HA, Ballinger C (2015). The causes of falls: views of older people with visual impairment. Health Expectations.

[41] Tiesman H, Zwerling C, Peek-Asa C, Sprince N, Cavanaugh JE (2007). Non-fatal injuries among urban and rural residents: The National Health Interview Survey, 1997–2001. Injury Prevention.

[42] White UE, Black AA, Wood JM, Delbaere K (2015). Fear of falling in vision impairment. Optometry and vision science.

[43] Li F, McAuley E, Fisher KJ, Harmer P, Chaumeton N, Wilson NL (2002). Self-efficacy as a mediator between fear of falling and functional ability in the elderly. Journal of aging and health.

[44] Jefferis BJ, Iliffe S, Kendrick D (2014). How are falls and fear of falling associated with objectively measured physical activity in a cohort of community-dwelling older men?. BMC Geriatrics.

[45] Legood R, Scuffham P, Cryer C (2002). Are we blind to injuries in the visually impaired? A review of the literature. Injury prevention.

